# Platelet-Rich Fibrin Synthetic Bone Graft Enhances Bone Regeneration and Mechanical Strength in Rabbit Femoral Defects: Micro-CT and Biomechanical Study

**DOI:** 10.3390/jfb16080273

**Published:** 2025-07-28

**Authors:** Yu-Kuan Lin, Hsuan-Wen Wang, Po-Kuei Wu, Chun-Li Lin

**Affiliations:** 1 Department of Biomedical Engineering, National Yang Ming Chiao Tung University, Taipei 112304, Taiwan; nickhsnu@gmail.com (Y.-K.L.); 411918@gmail.com (H.-W.W.); 2 Division of Joint Reconstruction, Department of Orthopaedics, Taipei Veterans General Hospital, Taipei 112, Taiwan; pkwu@vghtpe.gov.tw; 3 Medical Device Innovation & Translation Center, National Yang Ming Chiao Tung University, Taipei 112304, Taiwan

**Keywords:** platelet-rich fibrin, synthetic bone graft, sticky bone, compression test, micro-CT

## Abstract

This study evaluated the bone regeneration effect and mechanical properties of “Sticky bone”, a mixture of platelet-rich fibrin (PRF) and synthetic bone grafts (SBGs), in the repair of large femoral bone defects in rabbits. Eighteen New Zealand white rabbits were included and randomly divided into a Sticky bone group and an SBG alone group. Bone graft samples were collected and analyzed at 4, 8, and 12 weeks after surgery. Micro- computed tomography (CT) analysis showed that the amount of the Sticky bone group in the grayscale ranges of 255–140 (highly mineralized tissue or unabsorbed bone powder) and 140–90 (representing new cancellous bone) was higher than that of the SBG group at each time point and decreased with the number of weeks. The compression strength test showed that the average compression strength of the Sticky bone group reached 5.17 MPa at the 12th week, which was 1.62 times that of the intact bone (3.19 MPa) and was significantly better than that of the SBG group (about 4.12 MPa). This study also confirmed for the first time that the use of a new polyethylene terephthalate (PET) blood collection tube to prepare PRF can stably release key growth factors such as platelet-derived growth factor-BB (PDGF-BB) and vascular endothelial growth factor (VEGF), which are conducive to early bone vascularization and cell proliferation. In summary, Sticky bone has the potential to promote bone formation, enhance tissue integration and mechanical stability, and can be used as an effective alternative material for repairing large-scale bone defects in clinical practice in the future.

## 1. Introduction

Synthetic bone grafts (SBGs) are widely employed in the clinical treatment of various bone defects, including post-traumatic bone loss, reconstruction following tumor resection, spinal fusion procedures, joint revision surgeries, and craniofacial reconstruction [1,2]. Among the most commonly used SBG materials are calcium phosphate ceramics—such as hydroxyapatite (HA) and β-tricalcium phosphate (β-TCP)—which exhibit excellent biocompatibility and osteoconductive properties [3,4,5]. These materials effectively fill bone voids and provide initial mechanical stability, thereby supporting new bone formation and promoting the healing process [3,4,5].

Nevertheless, current SBGs possess osteoconductive properties but lack effective osteoinductive capability. Commercially available SBGs—typically in powder or cubic forms with highly porous architectures—often fail to achieve a tight fit within bone defects or the internal implant spaces. This loose interface between the graft material and the surrounding bone or implant surface can result in delayed healing or even non-union [5,6,7,8]. Consequently, the mechanical performance of these grafts is often suboptimal, providing insufficient initial stability—particularly in large defects or areas exposed to high mechanical stress. Complications such as particle micro-motion or irregular resorption rates are commonly observed. Moreover, due to the chemical nature of metallic salts, many SBGs degrade more rapidly than the surrounding bone can regenerate [9,10,11,12]. This mismatch may compromise the structural integrity of the healing site, increasing the risk of biomechanical failure, including bone cracking or fracture under physiological loads. As such, the use of SBGs alone remains inadequate to fully address the multifaceted clinical challenges of bone defect repair, particularly in enhancing material bioactivity and accelerating bone regeneration.

To address the limitations of SBG powders—particularly in their lack of viscosity and osteoinductive capacity—clinical practice has increasingly adopted the strategy of combining SBG powders with platelet-rich fibrin (PRF). PRF is a natural fibrin matrix derived from autologous blood through centrifugation. Compared to the traditional silica-coated tubes used for centrifugation, polyethylene terephthalate (PET)-based centrifuge tubes have advantageous properties, such as high transparency, good mechanical strength, chemical stability, and biocompatibility. PRF is rich in growth factors such as PDGF, transforming growth factor-β (TGF-β), and VEGF, as well as immune cells [13,14,15,16,17,18]. It offers several advantages, including excellent biocompatibility, ease of preparation, and the absence of immunogenic risk. Clinical studies have demonstrated that mixing PRF with bone graft powder to create “Sticky bone” can significantly enhance bone regeneration outcomes [5]. PRF enables the sustained release of growth factors, thereby improving the osteoinductive potential of the graft material, promoting angiogenesis, and accelerating the bone defect healing process. The inherent viscosity of PRF improves the SBG powders’ handling properties, making them easier to mold and more effective in filling complex or irregularly shaped bone defects [19,20,21,22].

Although “Sticky bone”—a composite of PRF and SBG powder—shows promising clinical potential, its ability to withstand high mechanical loads and meet the long-term requirements of large-scale bone defect repair remains uncertain due to its inherently weak mechanical properties [23,24,25]. Furthermore, the effects of Sticky bone on the rate of new bone formation and the resorption dynamics of the SBG powder have not been fully elucidated. Therefore, the objective of this study is to evaluate and compare the osteoinductive capacity and biomechanical performance of SBG powder alone versus Sticky bone (SBG powder combined with PRF). These materials were implanted into femoral condyle defects in rabbits, with specimens harvested at 4, 8, and 12 weeks post-implantation. The samples were assessed for new bone formation rate through micro-CT scans and compressive strength tests to determine the efficacy of bone induction and the extent of biomechanical enhancement.

## 2. Materials and Methods

This study utilized 18 healthy New Zealand white rabbits (average age: 12 months; average weight: 3.5 kg), in which either SBGs + PRF (Sticky bone) or SBGs alone were implanted into the distal femoral condyle. The animals were euthanized at 4, 8, and 12 weeks post-operation to evaluate new bone formation and mechanical strength. The rabbits were randomly assigned to two experimental groups: Group 1 received Sticky bone, and Group 2 received only SBGs, with three rabbits in each group. The right femur of each rabbit was designated for surgical intervention and subsequent bone regeneration assessment. Additionally, six left femurs from the total cohort of 18 rabbits were randomly selected and designated as a control group for comparative analysis. At each time point (4, 8, and 12 weeks), the femoral segments from both the right (experimental) and left (control) sides were harvested post-sacrifice. Micro-CT imaging was performed to visualize the bone-filling area, and quantitative bone quality assessments were conducted by applying grayscale threshold segmentation to distinguish residual SBG powder, newly mineralized bone, and soft or non-mineralized tissues. Following imaging, bone specimens were collected using a bone harvesting instrument, and compressive strength testing was carried out in accordance with the ISO 5833 Annex E protocol [26].

### 2.1. PRF Preparation and Validation

**Figure 1:**
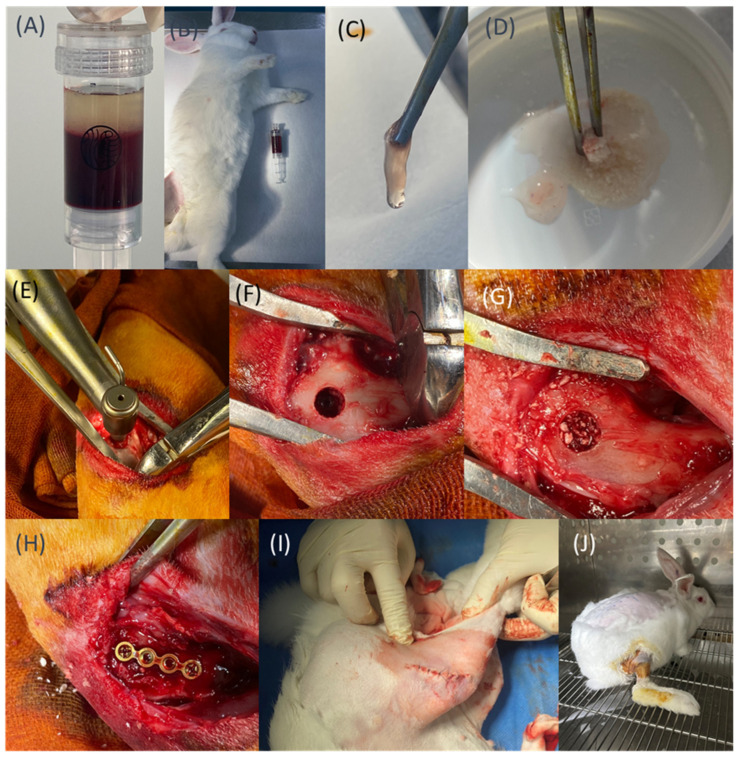
(**A**) The novel PET-based blood collection tube features a built-in negative-pressure mechanism. (**B**,**C**) Preparation of PRF. (**D**) Preparation of Sticky bone. (**E**–**G**) Drilling and filling of defects with different materials according to group assignments. (**H**) Placement of the fixation plate. (**I**) Closure of the muscle layer. (**J**) Postoperative care and observation.

Because rabbit blood samples are usually obtained from ear capillaries, traditional manual aspiration methods using fine butterfly needles connected to standard syringes typically encounter significant challenges, including difficulty in smoothly drawing blood and prolonged collection times. To address these issues, and to obtain high-quality PRF, a novel negative-pressure syringe centrifugation system (RYZ Care Platelet Plasma Separation System, National Yang Ming Chiao Tung University, Taipei, Taiwan), constructed of polyethylene terephthalate (PET), was utilized in this study. The innovative design of this PET-based blood collection tube incorporates a negative-pressure mechanism, facilitated by a silicone-sealed plunger in a specially designed 15 mL syringe that can be securely locked to maintain consistent negative pressure (Figure 1A). It is also compatible with standard double-ended needles and fine butterfly micro-needles, significantly enhancing the efficiency and ease of venous blood collection from rabbit ear capillaries, which then can be centrifuged directly in the centrifuge.

Unlike traditional silica-coated tubes, these PET-based centrifuge tubes eliminate the potential risk of silica contamination. Their negative-pressure mechanism also ensures smooth, efficient blood sample aspiration. After collection, the blood was immediately centrifuged (400× *g*, 12 min, S300T centrifuge, Kubota, Japan) and allowed to rest for 10–15 min, promoting the formation of a stable fibrin clot. The resulting PRF clot was subsequently separated from the underlying red blood cell layer and could be mixed with a commercial SBG.

To initially validate the biological efficacy of our novel PET-based PRF preparation tubes, an enzyme-linked immunosorbent assay (ELISA) was performed to quantify the contents of three key growth factors (VEGF: vascular endothelial growth factor, PDGF-BB: platelet-derived growth factor-BB, and IGF-1: insulin-like growth factor-1), comparing PRF samples prepared using the PET-based tubes with those from conventional silica-coated glass tubes (Vacutainer Plus, BD, Franklin Lakes, NJ, USA). Venous blood was collected from 10 healthy volunteers, and PRF was prepared according to identical protocols for both tubes. This study was approved by the Institutional Review Board of National Yang Ming Chiao Tung University (IRB No. NYCU113043AEF).

The resulting PRF samples were incubated in 6-well plates containing 5 mL of Dulbecco’s Modified Eagle Medium (DMEM) at 37 °C under 5% CO_2_ conditions for 14 days. Culture media were collected at days 1, 7, and 14 and then stored at −80 °C prior to ELISA analysis using commercial assay kits (Elabscience, Houston, TX, USA). Growth factor concentrations were determined at each time point, and cumulative amounts were calculated to evaluate the overall biological activity. VEGF data were obtained from our previous study [27], whereas PDGF-BB and IGF-1 levels were newly measured in this study and subsequently compared.

### 2.2. In Vivo Sticky Bone and SBG Implantation Experiment

All in vivo experimental procedures were conducted in accordance with protocols approved by the Institutional Animal Care and Use Committee of Master Laboratory Co., Ltd. (Approval No. 24T10-0202). All procedures adhered to the ARRIVE (Animal Research: Reporting of In Vivo Experiments) guidelines. Efforts were made to minimize animal usage and alleviate pain. The distal femoral condyle was selected as the implantation site due to its accessibility for bone implant experiments.

The in vivo procedure is summarized in Figure 1B–J. All animals fasted for 12 h before surgery. Anesthesia was induced via intramuscular injection of Zoletil (10 mg/kg) and xylazine (10 mg/kg), while meloxicam (1 mg/kg) was administered as needed for pain management. Once adequate sedation was achieved, venous blood was collected from the marginal ear vein using PET-based negative-pressure centrifuge tubes. PRF was subsequently prepared following the previously established protocol (Figure 1B,C). A total of 1.0 mL of PRF was obtained and thoroughly mixed with 1.0 g of synthetic bone graft material (ABCcolla^®^ Collagen Bone Graft; ACRO Biomedical Co. Ltd., Taiwan) at a 1:1 ratio, forming a cohesive Sticky bone graft with a total weight of approximately 2 g for implantation (Figure 1D) [6].

Each rabbit was placed in a supine position, and a 3–4 cm longitudinal skin incision was made on the lateral side of the hind knee to expose the lateral femoral condyle. A bone-holding clamp equipped with a directional guide was used to align the drilling axis. A bone tunnel with a diameter of 6 mm was created, stopping upon reaching the contralateral cortical bone, and without penetrating it (Figure 1E,F). Following drilling, the defect was filled with different materials based on the group assignment: Group 1 received Sticky bone, while Group 2 received SBGs alone (Figure 1G). To prevent early graft material dislodgement during the initial bone healing, a 1.2 mm straight titanium bone plate (cut to 4 holes from a 24-hole plate; Tedrill CMF System, Taipei, Taiwan) was used to cover the defect site (Figure 1H). The muscle layer was closed using absorbable sutures, while non-absorbable sutures were used for skin closure (Figure 1I).

Postoperatively, intramuscular injections of enrofloxacin (5 mg/kg) were administered for five consecutive days to prevent infection. The animals were housed in a monitored facility, where their food intake, mobility, and overall behavior (e.g., ability to stand and walk) were carefully observed (Figure 1J). Supportive care or euthanasia was provided if any signs of postoperative complications were noted. At the predetermined time points (4, 8, and 12 weeks), the animals were euthanized. Anesthesia was first administered to ensure full unconsciousness, followed by euthanasia using CO_2_ gas. After confirming death (approximately 5–6 min later), bone harvesting procedures were performed. The femoral segments from both the left and right hind limbs of each rabbit were collected, with the surrounding soft tissues around the distal femur carefully removed. The samples were then fixed in 4% formaldehyde for preservation prior to further analysis.

### 2.3. Micro-CT Scan and Image Processing

**Figure 2:**
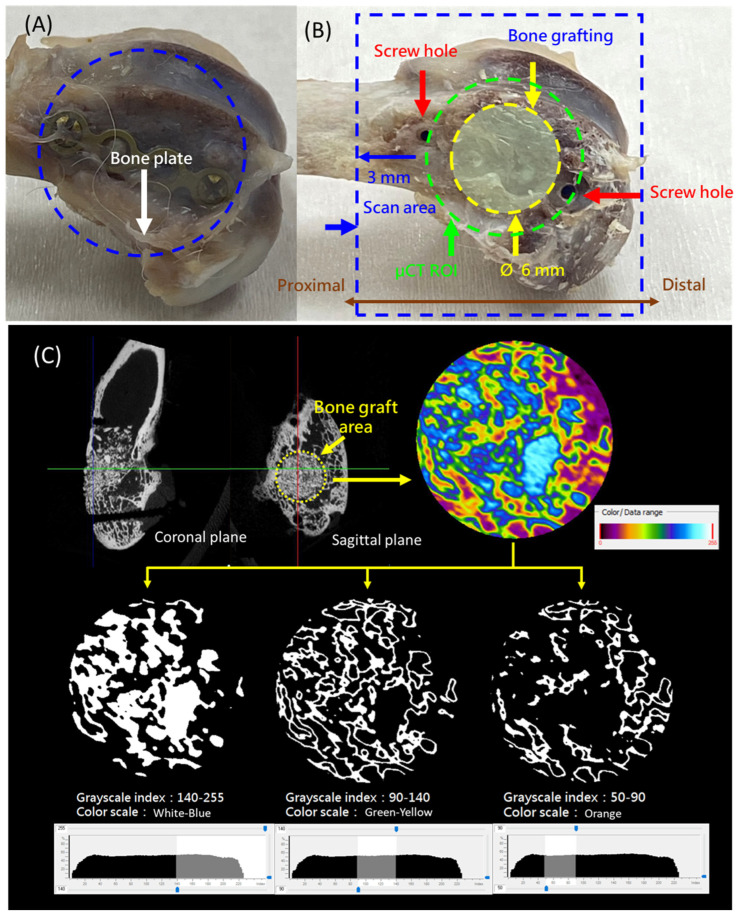
(**A**) The removed rabbit distal femur with the fixation plate attached. (**B**) Definition of the scanning range and a cylindrical region of interest (ROI) measuring 6 mm in diameter and 6 mm in height at the bone graft site. (**C**) Threshold values for micro-CT analysis: 140–255 indicates residual synthetic bone graft (SBG) or newly mineralized bone, 90–140 represents newly formed bone, and 50–90 corresponds to soft tissue or other non-bony components.

Within three days after harvesting, the femoral samples were submitted for micro-CT scanning. Prior to scanning, the temporary fixation components—including the covering bone plate and screws—were carefully removed (Figure 2A). The original screw holes served as anatomical landmarks for scan alignment. As shown in Figure 2B, the scanning range was defined starting from a point 3 mm proximal to the proximal screw hole and extended distally to the end of the femoral segment. For image analysis, a cylindrical region of interest (ROI) measuring 6 mm in diameter and 6 mm in height was defined at the bone graft site. Micro-CT imaging was performed using a Skyscan 1076 system (Bruker, Kontich, Belgium), with the scanning parameters set to a resolution of 18 µm at 90 kV.

The CT images were analyzed based on grayscale thresholding, with reference ranges established as described in the literature [28]. The threshold values were defined as follows: 140–255 indicated residual SBG or newly mineralized bone, 90–140 represented newly formed bone, and 50–90 corresponded to soft tissue or other non-bony components (Figure 2C). These thresholds were used to guide the subsequent quantitative analysis.

For each sample, the total tissue volume (TV) and bone volume (BV) within the region of interest were calculated. The percent bone volume (BV/TV%) was determined to evaluate the extent of bone regeneration.

### 2.4. Mechanical Testing

**Figure 3:**
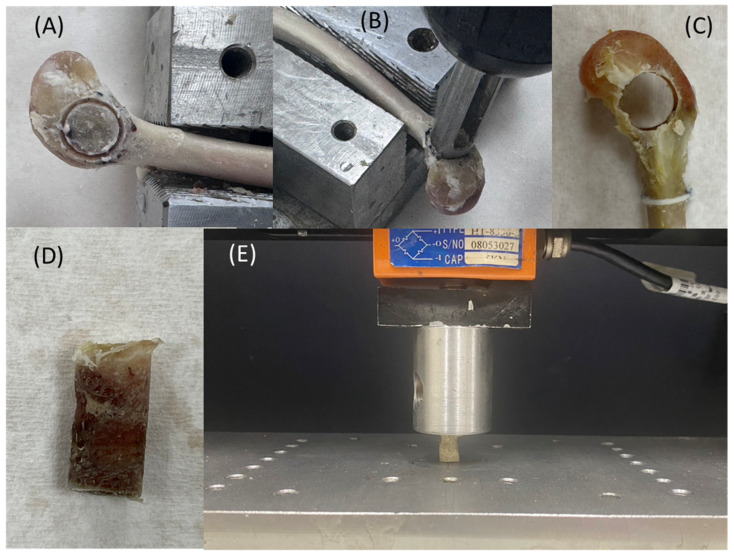
(**A**) Definition of the bone extraction region. (**B**) Custom-designed coring tool. (**C**) Bone core extracted from the rabbit femoral condyle, with actual dimensions measured. (**D**) Extracted bone sample. (**E**) Illustration of the compressive testing setup.

Following micro-CT scanning, destructive testing was conducted to evaluate the compressive strength of the regenerated bone. The bone extraction region was first defined using the two screw holes as anatomical landmarks (Figure 3A). A straight line was drawn between the centers of the two holes. The midpoint was used as the center of a circular region with a radius of 4 mm, marking the area for sample extraction.

A custom-designed coring tool (outer diameter Ø 9 mm, inner diameter Ø 8 mm) was used to extract the bone cylinder (Figure 3B). The coring procedure was monitored to ensure correct alignment with the planned extraction site. The resulting cylindrical bone sample had an approximate diameter of 8 mm. The actual dimensions (diameter and height) were measured using a Vernier caliper and recorded (Figure 3C).

Mechanical testing was performed using a universal testing machine (HT-2402EC, Hung Ta Instrument Co., Ltd., Taichung, Taiwan). The testing protocol was adapted from the ISO 5833 Annex E standard for bone cement compressive testing. The test setup is illustrated in Figure 3D,E. Compression was applied at a constant displacement rate of 20 mm/min, and the peak load was recorded. Compressive stress (σ) was calculated using the formula σ = F/A, where F is the maximum force and A is the bone sample’s cross-sectional area.

### 2.5. Statistical Analysis

All analyses were performed using IBM SPSS Statistics 29.0 (IBM, Armonk, NY, USA). The percent bone volume (BV/TV%), grayscale range, and compressive strength test results were analyzed using the Kruskal–Wallis H test, which is suitable for the relatively small sample size in our study; *p* < 0.05 was considered statistically significant.

## 3. Results

**Figure 4:**
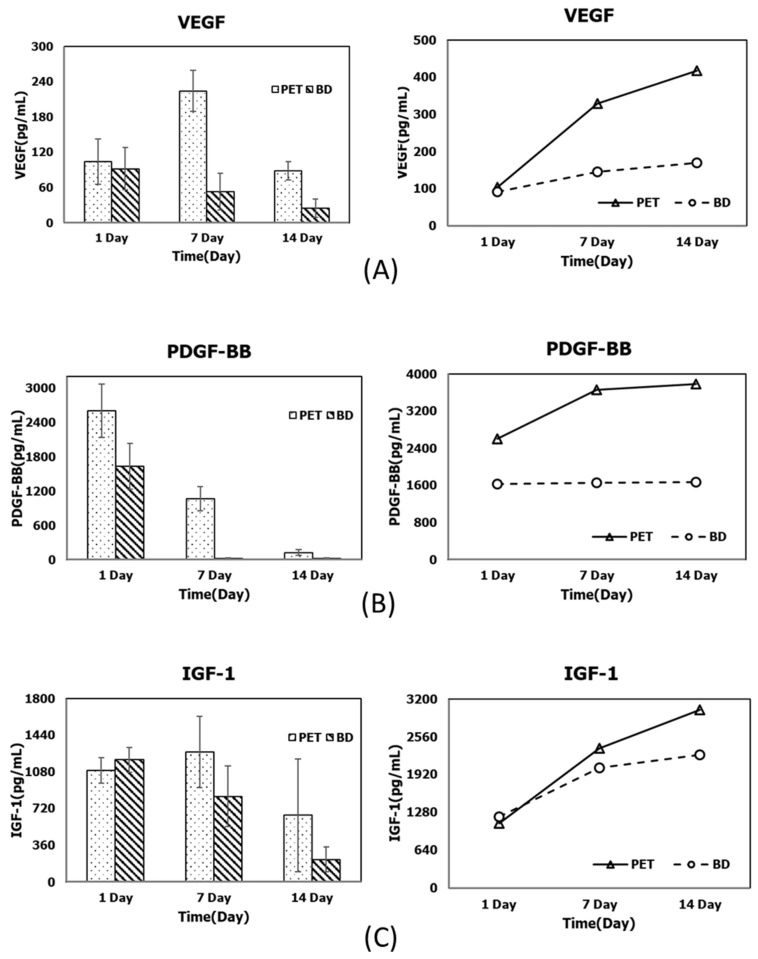
Comparison of PRF-derived growth factor release profiles prepared using the novel PET-based tubes and conventional silica-coated centrifuge tubes at different time points (1, 7, and 14 days) (**left**), and the corresponding cumulative values (**right**) for (**A**) VEGF, (**B**) PDGF-BB, and (**C**) IGF-1.

This study compared the PRF growth factor release prepared using the new PET-based and conventional silica-coated glass centrifuge tubes. The PET-based tubes performed well in the key growth factors that promote bone regeneration. Among them, the cumulative release of VEGF and PDGF-BB at individual time points in the early and mid-to-late stages (7–14 days) was significantly higher than for the silica-coated tubes (*p* < 0.05), as shown in Figure 4, indicating that PET-based blood tubes have obvious advantages in promoting neovascularization and cell proliferation in the early bone regeneration stages. In addition, in terms of the release of another important osteogenic factor, IGF-1, although the PET-based tubes were slightly inferior to the silica-coated tubes on the first day, the cumulative release increased rapidly from 7 to 14 days, and the final effect was close to that of the silica-coated tubes. This result indicates that the performance of the PET-based tubes in this key factor also reached a considerable level (Figure 4).

**Figure 5:**
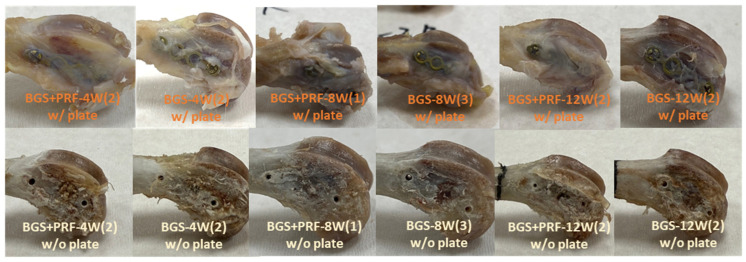
Gross appearance of the distal femoral end before (upper) and after (lower) removal of the fixation plate at 4, 8, and 12 weeks for the SBG + PRF and SBG groups. “SBG + PRF-XW(Y)” indicates the SBG + PRF group at X weeks, with Y representing the specific sample number as a representative example.

The upper and lower parts of Figure 5 show the appearance of the distal femoral end before and after the fixation plate was removed, respectively. At the same time point, no noticeable differences in shape or repair area were observed between the Sticky bone and SBG groups based on gross visual examination. However, the lack of a standardized quantitative scoring method must be acknowledged as a limitation. In comparisons at different time points, we found no increase in the density, tissue color, or repair degree of the bone defect regardless of whether the SBGs were mixed with PRF or not. In our study, there were no rabbits with any complications, such as fracture over the femur bone, superficial or deep infection, or death.

**Figure 6:**
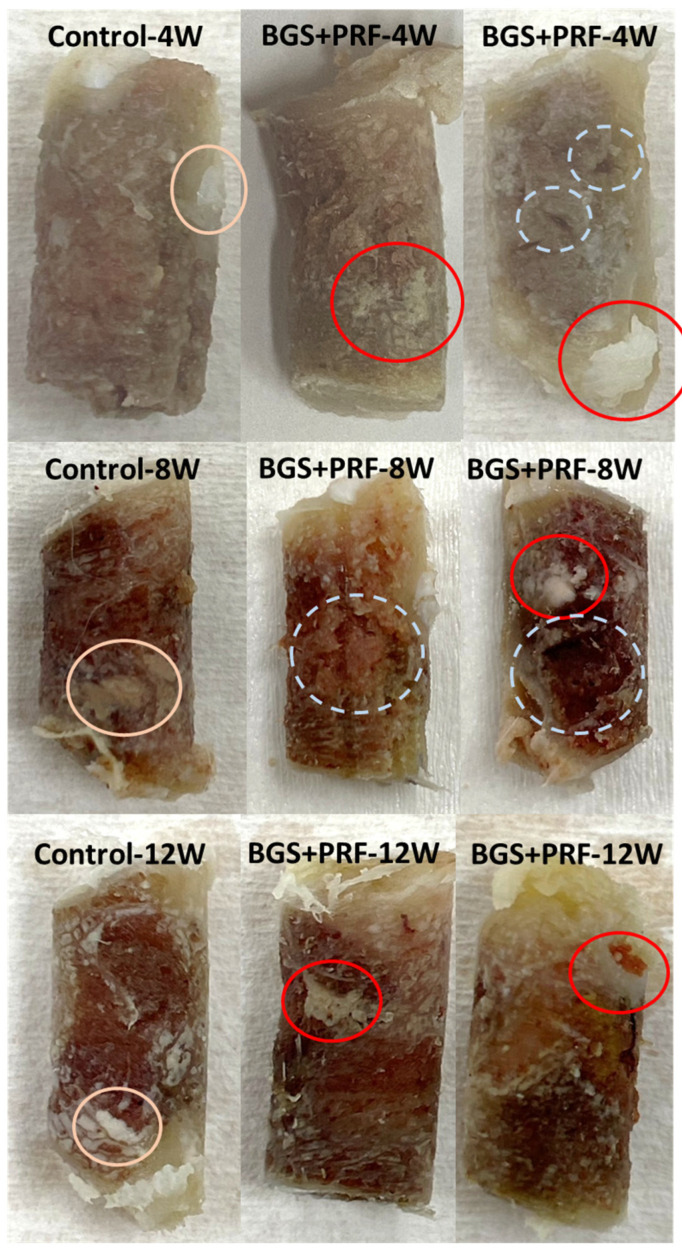
Comparison of the appearance of bone extraction regions under different conditions. From left to right: control, SBG + PRF (Sticky bone), and SBG groups. From top to bottom: observation at 4, 8, and 12 weeks, respectively. Pink circles: bone marrow tissue or fat, indicating normal bone tissue structure. Red circles: residual SBG powder granules. White dotted circles: regions of incomplete bone regeneration (e.g., voids or cavities).

Figure 6 shows a comparison of the appearance of the bone extraction regions under different conditions. In the intact bones (control group), regardless of the time point, the appearance showed no obvious defects, and the superior part was obviously the cortical bone region. The white parts within the yellow circle are the bone marrow tissue or fat from the inside of the bone, which is a presentation of the normal bone tissue structure. When Sticky bone and SBGs were used alone, artificial bone powder structures were found to be undegraded at each time point, with a white block shape and granular residues appearing within red circles. SBGs were more obvious than Sticky bone. At 4 and 8 weeks, the bone was not fully grown, and different-sized pores were found within the white dotted circles. At 12 weeks, the appearance of the Sticky bone and SBG groups tended to be stable and showed fewer pores.

**Figure 7:**
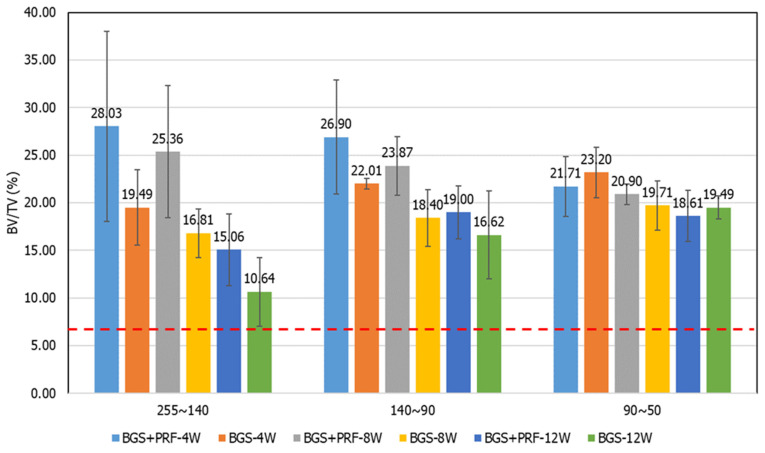
Bone volume fraction (BV/TV) results for each group at different time points, analyzed by grayscale ranges: (**A**) 255–140, (**B**) 140–90, and (**C**) 90–50. The red dotted line represents the BV/TV value of intact bone (7.5%).

In the micro-CT analysis, the bone volume ratio (BV/TV) results from each group at different time points and grayscale ranges are shown in Table 1 and Figure 7. From the contralateral intact bone reference values, it can be seen that the average BV/TV ratio of the three grayscale ranges was about 7.5%, which means that the distribution of cortical bone, cancellous bone, and non-bone tissue in normal bone tissue is relatively balanced. In contrast, the BV/TV values of the defect areas were much higher than the intact reference values, reflecting the presence of residual artificial bone powder or the formation of new bone tissue.

In the grayscale range of 255–140 (representing residual graft or highly mineralized bone), the Sticky bone group exhibited higher BV/TV values than the SBG group at all time points, although only the 8-week comparison reached statistical significance (*p* = 0.045), indicating the potential of PRF to promote early bone regeneration and mineralization. In the 140–90 grayscale range (representing newly formed cancellous bone), the Sticky bone group consistently demonstrated higher mean values than the SBG group, suggesting that PRF may enhance early-stage new bone formation, although these differences were not statistically significant. In the 90–50 range (representing low-density or un-mineralized tissue), no significant differences were observed between groups, and a high proportion of fibrous or immature tissue remained in the defect areas at all time points, indicating incomplete bone transformation.

**Figure 8:**
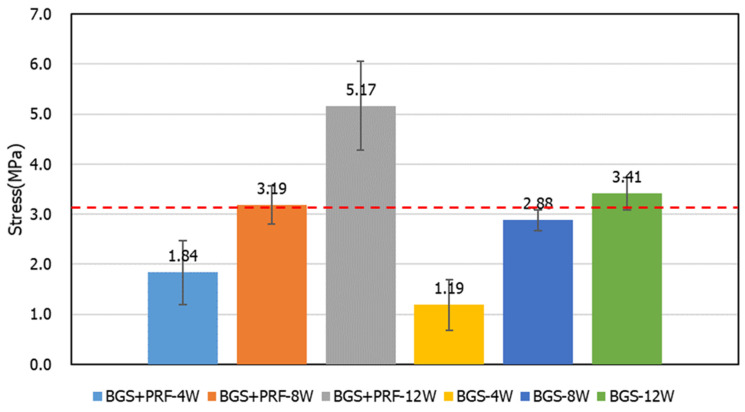
Compressive strength test results for different groups at 4, 8, and 12 weeks. The average compressive strength of the control group was approximately 3.19 MPa.

For the compression strength test (Figure 8), it can be seen that the compression strength increased over time, but with only the Sticky bone group at 12 weeks showing statistical significance (*p* = 0.0495). The average strength of the control group was about 3.19 MPa, while the compression strength of the Sticky bone group and the SBG group increased obviously with time. At 8 weeks, the compression strength of the Sticky bone group was equivalent to that of the control group. At 12 weeks, the compression strength of the Sticky bone group and the SBG group was higher than that of the control group. The strength of the Sticky bone could reach about 1.62 times that of the intact bone.

## 4. Discussion

This study verified the effectiveness of the newly developed negative-pressure PET-based centrifuge tubes in PRF preparation. Compared with conventional silica-coated blood centrifuge tubes, PET tubes have no risk of silicone coating contamination and have good negative-pressure design and sealing properties, effectively improving the blood collection efficiency and the stable release of growth factors. The ELISA results from this study showed that the cumulative release of PDGF-BB and VEGF from the 7th to the 14th day of PRF preparation using PET tubes was significantly higher than that with the glass tubes. IGF-1 showed a comparable release level in the later period, indicating that it has good biological activity and medium-term sustained action potential. Compared with the traditional PRF preparation method, which often leads to inconsistent quality due to differences in centrifuge tubes, the use of dedicated PET tubes can improve the consistency and standardization of PRF quality. For clinical units that need to prepare large quantities of PRF (such as dental implant centers, orthopedic surgery, etc.), this improved tube type provides a simple, safe, and scalable preparation method. It has the potential to be promoted as a commercial medical device to support the clinical implementation and routine application of PRF in the field of regenerative medicine. In addition, these novel PET-based tubes have been approved by the Taiwan FDA as a Class II medical device (registration number 008359), further ensuring their safety.

From the appearance comparison results in Figure 6, at 4 and 8 weeks, it was observed that the bone defects in both the Sticky bone and SBG groups had not been completely filled, and there were pores of different sizes until 8 weeks, indicating that the new bone was still in the process of activation and tissue filling at this stage. However, the appearance of both groups had obviously stabilized at 12 weeks, with fewer pores and a denser bone structure, reflecting that bone reconstruction had entered a mature stage. This result was consistent with the micro-CT analysis and compression strength testing, showing that Sticky bone can not only promote bone regeneration but also enhance tissue’s structural stability.

Micro-CT image analysis showed that percentage of BV/TV in the grayscale range of 140–90 (i.e., the new bone growth range) for the Sticky bone group was significantly higher than for the SBG group at all time points, indicating that PRF can effectively promote the regeneration and mineralization of cancellous bone. This finding is highly correlated with PDGF-BB, VEGF, and other molecules that promote cell proliferation and angiogenesis, which are abundant in PRF [29]. Based on our results, Sticky bone demonstrated the ability to accelerate early bone regeneration and mineralization, while also enhancing early load-bearing capacity. These features suggest that it may be suitable for clinical applications requiring rapid skeletal stabilization and early mechanical support, such as spinal fusion, initial stabilization of dental implants, and the repair of large bone defects. This approach has the potential to shorten the critical bone-healing period and reduce the risk of delayed union or non-union.

The grayscale range of 255–140 reflects incompletely absorbed SBG powder and highly mineralized new bone tissue. The results of this study showed that the Sticky bone group was still significantly higher than the SBG group at all time points, indicating that PRF may have the effect of stabilizing bone powder and reducing its initial rapid degradation. This is consistent with the physical properties of PRF itself, which forms a stable three-dimensional fiber network, encapsulates bone powder, and delays its exposure to body fluids and rapid absorption [14,30]. In many clinical cases, synthetic bone powder is easily absorbed prematurely in high-activity areas or areas with rich blood flow, resulting in insufficient skeleton and bone-healing failure. Sticky bone may be able to solve this clinical dilemma, prolong the bone powder’s existence time, and enable it to play a more complete bone guidance effect.

Although Sticky bone has a significant effect on promoting early bone formation, the data in the 90–50 grayscale range showed that, regardless of whether PRF was added, there was no obvious change trend at each time point and in SBGs alone, indicating that the tissue in some areas was still in the fibrous or soft tissue stage and had not yet been fully transformed into mature bone. This result shows that even though Sticky bone has an early induction effect, the maturation and remodeling of bone tissue in the middle and late stages may still be limited by the PRF release cycle (usually most factors are released within 7–14 days), and its long-term support effect is limited [23]. Clinically, this means that Sticky bone is suitable for accelerating early repair, but for lesions that require long-term support and complete bone integration it still needs to be combined with other bioactive materials or regenerative strategies (such as BMP-2 or mesenchymal stem cells).

The compression strength test results showed that the compression strength of the Sticky bone group reached 3.1 MPa at week 8, which was not higher than that of the intact bone group. At week 12, it increased to about 5.1 MPa, which was 1.62 times that of the control group and better than that of the simple SBG group. This proves that Sticky bone can effectively build a stable, load-bearing skeleton after material integration and bone tissue reconstruction. Its high initial adhesion and osteoinduction ability allow the material to provide mechanical stability at an early stage, making it particularly suitable for applications such as long bone shafts, subarticular bone defects, and craniofacial support areas that require early stability. Clinically, this can not only shorten the patient’s period of reconstruction but also reduce the risk of collapse or reoperation of the reconstructed area.

This study has several limitations. First, the relatively small sample size (n = 3 per group per time point) reduced its statistical power and may have increased the data variability. Although we employed non-parametric statistical methods such as the Kruskal–Wallis H test, which is suitable for small sample sizes, future studies with larger cohorts are warranted to confirm and strengthen our findings. Second, histological or immunohistochemical analyses were not performed. While micro-computed tomography (micro-CT) and compressive strength testing provided valuable insights into bone regeneration and mechanical performance, histological evaluation remains the gold standard for confirming tissue quality, cellular integration, angiogenesis, and inflammatory status. However, due to the limited number of samples and the destructive nature of histological processing, performing such analysis would have prevented us from assessing the temporal relationship between bone formation and mechanical strength. To preserve the integrity of the samples for biomechanical testing, we adopted a non-destructive approach using micro-CT imaging to assess bone structure and regeneration.

The effectiveness of micro-CT for evaluating bone formation has been demonstrated in several studies, and prior research has shown a strong correlation between micro-CT and histological findings [23,24,25]. For instance, Lappalainen et al. used micro-CT to monitor calvarial bone healing in rabbits, highlighting its excellent capacity to visualize bone architecture [23], while Ilan et al. demonstrated high consistency between histological and micro-CT analyses in evaluating new bone formation [24]. Nonetheless, micro-CT has inherent limitations. Although grayscale segmentation enables differentiation between residual synthetic bone grafts (SBGs), newly mineralized bone, and low-density or non-mineralized tissues, it cannot directly identify microstructures or biological responses such as osteocyte activity, bone–material interface integration, fibrotic tissue, or inflammation. Some high-grayscale regions may contain both unabsorbed SBG and early-stage mineralized bone, which are difficult to distinguish based solely on imaging. Therefore, future studies should incorporate histological and immunohistochemical staining techniques (e.g., H&E, Masson’s trichrome, TRAP, or osteocalcin) to more accurately assess bone tissue quality, verify the mechanism of PRF at the cellular level, and build a more comprehensive framework for evaluating scaffold performance and bone-healing outcomes.

Based on our results, this study confirms that Sticky bone has better advantages in accelerating new bone formation, stabilizing the absorption rate of SBG powder, and improving the mechanical strength of the reconstruction area. In addition, PRF is autologous, and it has a low preparation cost and extremely low immunogenicity, indicating that Sticky bone is a high-potential, standardized, and clinically ready bone regeneration combination material. In the future, the mixing ratio of PRF and SBG could be further optimized or sustained-release carriers (such as gelatin microspheres) could be combined to prolong the release time of growth factors and improve the bone maturation rate in the middle and late stages. In addition, it is also recommended to combine histological staining with molecular analysis to verify the effects of PRF on osteocyte differentiation and trabecular bone arrangement, thereby further supporting its feasibility as a standard protocol for clinical implantation.

## 5. Conclusions

This study confirmed that Sticky bone, made by combining PRF and SBG powder, can effectively promote bone regeneration, tissue mineralization, and higher compressive strength and mechanical stability in large-scale bone defects in rabbit femurs. Its stimulatory mechanism may be related to the sustained release of growth factors contained in PRF. This result shows that Sticky bone has good clinical application potential and is particularly suitable for the treatment of bone defects that require early stabilization and rapid healing. However, more research is needed to clarify the PRF release cycle and long-term support effect in Sticky bone.

## Figures and Tables

**Table 1:** The values of BV/TV% for different gray threshold ranges at 4, 8, and 12 weeks for the experimental and control groups.

	Group 1 (PRF + SBG) (Sticky Bone)				Group 2 (SGB)				Kruskal-Wallis H Test
4 Weeks (BV/TV%)									
Gray threshold values	No. 1	No. 2	No. 3	Average	No. 1	No. 2	No. 3	Average	*p* Value
Residual SBG or newly mineralized bone (255~140)	27.49	38.27	18.34	28.03	19.15	15.70	23.62	19.49	0.275
Newly formed bone (140~90)	26.04	33.28	21.38	26.90	22.34	22.30	21.39	22.01	0.513
Soft tissue or other non-bony components (90~50)	19.41	20.43	25.30	21.71	25.37	24.00	20.24	23.21	0.513
8 Weeks (BV/TV%)									
Gray threshold values	No. 1	No. 2	No. 3	Average	No. 1	No. 2	No. 3	Average	*p* Value
Residual SBG or newly mineralized bone (255~140)	20.16	33.25	22.66	25.36	19.72	14.91	15.81	16.81	0.0495
Newly formed bone (140~90)	23.02	27.29	21.30	23.87	16.37	21.84	16.98	18.40	0.127
Soft tissue or other non-bony components (90~50)	21.97	20.94	19.78	20.90	17.23	22.44	19.45	19.71	0.513
12 Weeks (BV/TV%)									
Gray threshold values	No. 1	No. 2	No. 3	Average	No. 1	No. 2	No. 3	Average	*p* Value
Residual SBG or newly mineralized bone (255~140)	12.58	19.40	13.21	15.07	8.47	8.64	14.81	10.64	0.275
Newly formed bone (140~90)	17.51	17.25	22.23	19.00	16.65	11.97	21.23	16.62	0.275
Soft tissue or other non-bony components (90~50)	20.06	15.50	20.28	18.61	18.96	18.65	20.87	19.49	0.827
Control group									
Gray threshold values	No. 1		No. 2	No. 3		No. 4	No. 5	No. 6	Average
Residual SBG or newly mineralized bone (255~140)	8.97		8.16	5.40		3.52	7.65	6.77	6.75
Newly formed bone (140~90)	8.39		9.91	8.09		6.58	9.08	8.92	8.50
Soft tissue or other non-bony components (90~50)	8.57		10.06	8.59		8.39	9.19	8.96	8.96
							Total average		7.5

## Data Availability

The original contributions presented in this study are included in the article; further inquiries can be directed to the corresponding author.

## Abbreviations

The following abbreviations are used in this manuscript:

SBG	Synthetic bone graft
HA	Hydroxyapatite
β-TCP	β-Tricalcium phosphate
PRF	Platelet-rich fibrin
PET	Polyethylene terephthalate
ELISA	Enzyme-linked immunosorbent assay
VEGF	Vascular endothelial growth factor
PDGF-BB	Platelet-derived growth factor
IGF-1	Insulin-like growth factor
ROI	Region of interest
TV	Tissue volume
BV	Bone volume
